# The Relationships Among FoMO, Problematic Social Media Use, Alexithymia, and Psychiatric Symptoms in Adolescents

**DOI:** 10.1192/j.eurpsy.2026.10243

**Published:** 2026-07-31

**Authors:** N. Serdengeçti, A. B. Can Aydın, M. Yavuz

**Affiliations:** 1 Haydarpaşa Research and Training Hospital; 2 Seyrantepe Hamidiye Research and Training Hospital; 3 Istanbul University-Cerrahpaşa, İstanbul, Türkiye

## Abstract

**Introduction:**

As social media use continues to increase, particularly among adolescents, understanding its impact on mental health is becoming increasingly urgent.

**Objectives:**

This study explored the relationships between alexithymia, FoMO, excessive social media use, and psychiatric symptoms in a clinical adolescent sample, hypothesizing that alexithymia would be linked to higher FoMO and social media addiction, with internalising and externalising problems as potential mediators. It also examined whether these variables differed based on daily use of popular social media platforms.

**Methods:**

A total of 197 participants aged 14 to 18 years were recruited from child and adolescent psychiatric outpatient clinics in two public hospitals. Participants were interviewed by a child and adolescent psychiatrist and then asked to complete the Strengths and Difficulties Questionnaire, the Alexithymia Questionnaire, the Bergen Social Media Addiction Scale and the Fear of Missing Out Scale.

**Results:**

Mediation analyses revealed that while alexithymia significantly predicted social media addiction, it did not directly predict FoMO. Instead, the relationship between alexithymia and FoMO was fully mediated by internalizing and externalizing problems. In addition, externalising symptoms partially mediated the relationship between alexithymia and social media addiction.

**Image 1:**
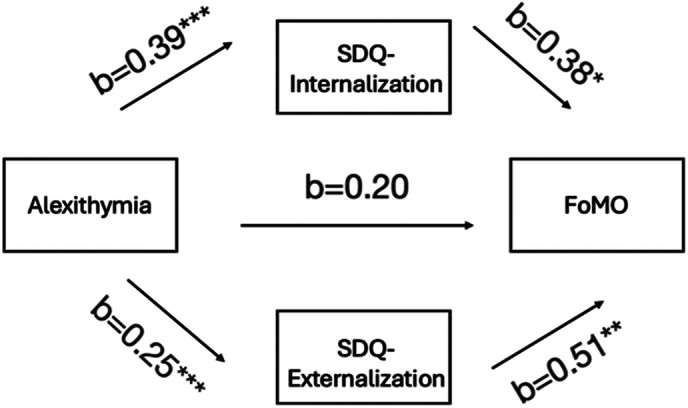

**Image 2:**
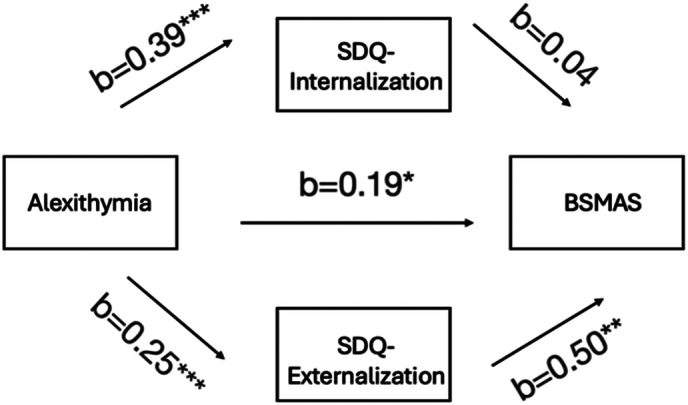

**Conclusions:**

These findings highlight the complex emotional and behavioural contexts underlying problematic social media use among adolescents and underscore the need for interventions that address emotional processing difficulties and comorbid psychopathology in this population.

**Disclosure of Interest:**

None Declared

